# Alteration of plasma phospholipids distinguish schizophrenic patients from controls: A targeted metabolomics study

**DOI:** 10.1192/j.eurpsy.2023.1282

**Published:** 2023-07-19

**Authors:** M. Naifar, A. Tebani, F. Ducatez, C. Pilon, T. Plichet, M. Maalej, W. Guidara, M. Maalej, F. AYADI, S. Bekri

**Affiliations:** 1 Biochemistry Laboratory “Molecular Basis of Human Diseases”, LR19ES13, Sfax Medicine College, University of Sfax, Sfax, Tunisia; 2 Department of Metabolic Biochemistry, UNIROUEN, INSERM U1245, CHU Rouen, Rouen University Hospital, Normandie University, Rouen, France; 3 Psychiatry C- Department, Hedi Chaker University Hospital, University of Sfax, Sfax, Tunisia

## Abstract

**Introduction:**

Schizophrenia (SCZ) is one of the most severe mental disorders. Several elements involved in pathogenesis have been characterized recently. However, tools for diagnosis and risk prediction are limited. Elucidation of the underlying genomic and molecular mechanisms of SCA remains a challenge.

**Objectives:**

In this study, we aimed to identify plasma biomarkers for SCZ using targeted metabolomics.

**Methods:**

All enrolled patients were drug-free for at least 3 months prior to admission. Plasma from 31 SCZ patients and 70 matched controls were analyzed using the LC/MS- Api 4000 QTrap Sciex. A total of 188 targeted metabolites, including 21 amino acids, 21 biogenic amines and 145 lipids or lipid-related metabolites were analyzed. All data modeling and analysis is done using MetaboAnalyst 5.0

**Results:**

There was no significant difference in the studied groups regarding BMI. Plasma Triglycerides, LDL-C, total proteins levels were significantly decreased in SCZ compared to controls. Heatmap identified 2 clusters with 25 significantly differentially expressed metabolites (FDR <0.05) between the drug-naïve group and the matched controls. The OPLS-DA score plot showed that the groups are clearly separated according to plasma phospholipids concentrations. Among these differential metabolites, the expression level of very long chain Phosphatidylcholines (PC 36 – PC p42) and acylcarnitines were significantly decreased in SCZ compared to controls, whereas sphingomyelin (SM) and lysoPC were significantly lower in drug-naive patients.

**Conclusions:**

In this study, we found that plasma phospholipids were significantly dysregulated in the SCZ patients and could be a promising pathway to explore SCZ.

**Disclosure of Interest:**

None Declared

